# Knowledge and Practices of Healthcare Providers Regarding Neonatal Transport in Eastern Morocco: A Cross-Sectional Study

**DOI:** 10.7759/cureus.107691

**Published:** 2026-04-25

**Authors:** Mohammed Ech-Chebab, Anass Ayyad, Hanae Bahari, Sahar Messaoudi, Rim Amrani

**Affiliations:** 1 Neonatology and Neonatal Intensive Care, Mohammed VI University Hospital, Oujda, MAR; 2 Mother and Child Health Laboratory, Mohammed First University, Faculty of Medicine and Pharmacy of Oujda, Oujda, MAR

**Keywords:** healthcare providers, hypothermia, knowledge and practices, morocco, neonatal care, neonatal transport

## Abstract

Background: Neonatal transport is a critical component of neonatal care, particularly in low-resource settings where inadequate transport conditions may contribute to increased neonatal morbidity and mortality. This cross-sectional study aimed to assess knowledge and practices related to neonatal transport among healthcare providers in Eastern Morocco.

Methods: This study included healthcare facilities providing care at different levels. A structured anonymous questionnaire created using Google Forms was distributed online. Data was collected between April 1 and April 9, 2026. Statistical analysis was performed using Microsoft Excel (Microsoft Corporation, Redmond, USA).

Results: A total of 77 healthcare providers participated in this study. Most participants correctly identified key aspects of neonatal transport, including neonatal stabilization (70/77, 90.9%), thermoregulation (75/77, 97.4%), and recognition of hypothermia (57/77, 74.0%). However, reported practices were less consistent. Only 40 (51.9%) participants consistently performed stabilization before transport, 35 (45.5%) ensured continuous monitoring, and 38 (49.4%) of the 77 participants systematically applied preventive measures against hypothermia. In addition, standard ambulances were the most commonly used means of transport (52/77, 67.5%), while only 20 (26.0%) reported using equipped ambulances.

Conclusion: Neonatal transport practices in Eastern Morocco remain inconsistent despite relatively high levels of knowledge among healthcare providers. This gap between knowledge and practice highlights the need for structured training programs and improved transport infrastructure to enhance neonatal outcomes in resource-limited settings.

## Introduction

Neonatal mortality remains a major global health concern, particularly in low- and middle-income countries, where it accounts for a substantial proportion of under-five mortality [1]. The quality of neonatal care, including safe and effective transport systems, plays a critical role in improving neonatal outcomes, especially for critically ill newborns requiring referral to higher level facilities [2].

Neonatal transport is a complex process that requires adequate stabilization before transfer, continuous monitoring during transport, and appropriate thermal protection. However, in many resource-limited settings, including Morocco, neonatal transport remains inadequately organized, with limited availability of specialized equipment and trained personnel. Consequently, transfers are often carried out under suboptimal conditions, sometimes without proper thermal support or monitoring.

Hypothermia is one of the most frequent and serious complications during neonatal transport and is strongly associated with increased morbidity and mortality [3,4]. Preventing hypothermia through appropriate thermal protection measures is therefore essential to ensure safe transport conditions. Despite its importance, data on neonatal transport practices and healthcare providers’ knowledge in Morocco remain scarce. In the Oriental region, where newborns are frequently transferred from peripheral and rural areas to tertiary care centers, transport-related challenges are particularly significant.

The present study aimed to assess the knowledge and self-reported practices related to neonatal transport among healthcare professionals working in the Oriental region of Morocco. Specifically, the objectives were as follows: (1) to evaluate the level of knowledge regarding key aspects of neonatal transport, including stabilization, thermoregulation, hypothermia prevention, and monitoring; (2) to describe current practices in neonatal stabilization, transport methods, thermal protection, and monitoring during transfer; and (3) to identify gaps between knowledge and practice that may inform future training and system improvements.

## Materials and methods

This was a cross-sectional descriptive study conducted in the Oriental region of Eastern Morocco. The study included healthcare facilities at different levels of care, including a tertiary university hospital, regional hospitals, provincial hospitals, and primary healthcare centers. The study targeted healthcare professionals involved in neonatal care and neonatal transport, including pediatricians, pediatric residents, general practitioners working in emergency settings, nurses, and midwives. Participants were required to be actively involved in neonatal care. Participation was voluntary and anonymous.

A convenience sampling method was used. Data were collected using a structured, self-administered questionnaire created using Google Forms. The questionnaire was distributed online through professional networks and social media platforms. Due to this distribution method, the total number of individuals who received the questionnaire could not be determined, and therefore, the response rate could not be calculated.

The questionnaire was developed based on a review of the literature and adapted to the local context. It consisted of three sections: (1) demographic characteristics, (2) knowledge assessment, and (3) practices related to neonatal transport. The knowledge section included 11 questions, with each correct answer awarded one point, resulting in a total possible score ranging from 0 to 11. The questionnaire was reviewed by neonatology specialists to ensure clarity and relevance; however, it was not formally validated. The full questionnaire used for data collection is provided in the Appendices.

Data were analyzed using descriptive statistics and presented as frequencies and percentages. Statistical analysis was performed using Microsoft Excel (Microsoft Corporation, Redmond, USA). Data collection was conducted from April 1 to 9, 2026. Participation was voluntary, and informed consent was obtained electronically from all participants before inclusion. The survey was anonymous, and no identifiable personal data were collected. The study was conducted in accordance with ethical standards for research involving human participants. Due to the observational and survey-based nature of the study, formal ethical approval was not required.

## Results

A total of 77 healthcare professionals participated in this study (Table 1). Participants included midwives (20, 26.0%), nurses (19, 24.7%), emergency physicians (14/77, 18.2%), pediatricians (11, 14.3%), and pediatric residents (8, 10.4%), while a smaller proportion consisted of obstetrics and gynecology residents (5, 6.5%). Participants were distributed across different healthcare settings: university hospitals (19/77, 24.7%), regional hospitals (8/77, 10.4%), provincial hospitals (14/77, 18.2%), urban health centers (15/77, 19.5%), rural health centers (16/77, 20.8%), and private sector facilities (5/77, 6.5%).

**Table 1:** Sociodemographic and professional characteristics of participants (n = 77)

Variable	Category	n	%
Profession	Pediatrician	11	14.3
	Pediatric resident	8	10.4
	Emergency physician	14	18.2
	Obstetrics and Gynecology resident	5	6.5
	Nurse	19	24.7
	Midwife	20	26.0
Workplace	University hospital (CHU)	19	24.7
	Regional hospital	8	10.4
	Provincial hospital	14	18.2
	Urban health center	15	19.5

In terms of professional experience, 41 (53.2%) participants had two to five years of experience, 20 (26.0%) had more than five years of experience, and 16 (20.8%) had less than two years of experience. Regarding involvement in neonatal transport, 40 (51.9%) of 77 participants reported frequent involvement, 24 (31.2%) reported occasional involvement, and 13 (16.9%) reported rare involvement. Notably, 73 (94.8%) participants reported not having received any formal training in neonatal transport.

Regarding knowledge of neonatal transport, 70 (90.9%) participants correctly identified newborn stabilization as the most important step before transport (Table 2). A total of 69 (89.6%) participants correctly identified the normal neonatal temperature range (36.5-37.5°C), and 57 (74.0%) participants correctly defined hypothermia as a temperature below 36.5°C. Almost all participants identified hypothermia as the most frequent complication during neonatal transport (75, 97.4%) and recognized appropriate thermal protection (incubator or skin-to-skin contact) as the best preventive measure (75, 97.4%). Furthermore, 74 (96.1%) participants correctly stated that monitoring during transport should include all vital parameters.

**Table 2:** Knowledge related to neonatal transport (n = 77)

Question	Correct answer	n	%
Most important step before transport	Stabilization	70	90.9
Normal neonatal temperature	36.5–37.5°C	69	89.6
Definition of hypothermia	<36.5°C	57	74.0
Most frequent complication	Hypothermia	75	97.4
Prevention of hypothermia	Adequate thermal protection	75	97.4
Monitoring during transport	All vital parameters	74	96.1
Ideal transport conditions	Equipped ambulance with trained staff	72	93.5
Blood glucose management	Checked and corrected if needed	75	97.4
Oxygen administration	If needed, with monitoring	75	97.4
Before transfer	Contact the receiving center	73	94.8

Regarding optimal transport conditions, 72 (93.5%) participants reported that neonatal transport should be carried out using a well-equipped ambulance with trained healthcare personnel. A total of 75 (97.4%) participants indicated that blood glucose should be checked before transport and corrected if necessary, and 75 (97.4%) stated that oxygen should be administered only when needed with appropriate monitoring (Table 2). Additionally, 73 (94.8%) participants emphasized the importance of contacting the receiving center before transfer, and 75 (97.4%) participants agreed that the newborn should be accompanied by trained healthcare personnel.

Despite these findings, reported practices were less consistent. Only 40 (51.9%) of the 77 participants reported always stabilizing the newborn before transport, whereas 36 (46.8%) reported doing so only sometimes, and 1 (1.3%) participant reported never performing stabilization (Table 3). Regarding transport methods, standard ambulances were the most commonly used means of transport (52/77, 67.5%), followed by equipped ambulances (20/77, 26.0%), while personal vehicles were rarely used (5/77, 6.5%).

**Table 3:** Practices related to neonatal transport (n = 77)

Variable	Category	n	%
Stabilization before transport	Always	40	51.9
	Sometimes	36	46.8
	Never	1	1.3
Transport method	Standard ambulance	52	67.5
	Equipped ambulance	20	26.0
	Personal vehicle	5	6.5
Prevention of hypothermia	Always	38	49.4
	Sometimes	36	46.8
	Never	3	3.9
Monitoring during transport	Always	35	45.5
	Sometimes	41	53.2
	Never	1	1.3

Preventive measures against hypothermia were always applied by 38 (49.4%) participants, while 36 (46.8%) reported applying them only sometimes, and 3 (3.9%) reported never applying them. Similarly, continuous monitoring of vital signs during transport was always performed by 35 (45.5%), whereas 41 (53.2%) participants reported doing so only sometimes, and 1 (1.3%) participant reported never performing monitoring (Table 3).

## Discussion

This study evaluated healthcare providers’ knowledge and self-reported practices regarding neonatal transport in the Oriental region of Morocco. The findings highlight a discrepancy between relatively high levels of knowledge on key aspects of neonatal transport and less consistent reported clinical practices, indicating important gaps in neonatal transport care.

Participants demonstrated high levels of knowledge regarding neonatal transport principles. Most participants correctly identified newborn stabilization as the most important step before transport (70/77, 90.9%) and recognized hypothermia as the most frequent complication during neonatal transport (75/77, 97.4%). These findings are consistent with previous studies conducted in similar settings, which reported satisfactory knowledge among healthcare providers involved in neonatal care [1,2]. Despite this, reported practices were less consistent. Only 40 (51.9%) of 77 participants reported always stabilizing newborns before transport, and continuous monitoring during transfer was consistently performed by only 35 (45.5%). This discrepancy between knowledge and practice has been widely reported in low- and middle-income countries and may reflect systemic and organizational constraints rather than lack of awareness alone [3,4].

A key finding of this study is the very low rate of formal training in neonatal transport, with 73 (94.8%) participants reporting no prior formal training. While this may contribute to variability in practices, the cross-sectional design of the study does not allow for causal inferences. Previous research has shown that structured training programs are associated with improvements in neonatal stabilization and transport quality [5,6].

Hypothermia remains a major concern during neonatal transport. It is one of the most frequent complications and is strongly associated with increased neonatal morbidity and mortality [7,8]. Although nearly all participants recognized its importance and identified appropriate preventive measures (75/77, 97.4%), only 38 (49.4%) reported consistently applying these measures in practice. This gap may reflect limited access to appropriate equipment, such as transport incubators, or challenges in implementing thermal protection strategies, particularly in peripheral and rural settings [9].

Another important finding is the predominant use of standard ambulances (52/77, 67.5%), with limited availability of adequately equipped neonatal transport systems (20/77, 26.0%). This highlights infrastructural challenges in the organization of neonatal transport services. Similar findings have been reported in other developing countries, where neonatal transport is often non-specialized and associated with increased risk of complications [10].

Encouragingly, most participants recognized the importance of communication with the receiving center before transfer (73/77, 94.8%) and the need for trained personnel to accompany the newborn (75/77, 97.4%). These elements are essential components of safe neonatal transport and are strongly recommended in international guidelines [11].

Taken together, these findings suggest that improving neonatal transport in this setting requires a multifaceted approach, including the implementation of structured training programs, strengthening of transport systems, and improved availability of appropriate equipment and monitoring tools. However, these conclusions should be interpreted as hypothesis-generating rather than definitive. Such interventions may contribute to reducing neonatal morbidity and mortality and align with global strategies aimed at improving neonatal survival [12-15].

This study has several limitations. First, the use of self-reported data may introduce reporting and social desirability bias, potentially leading to overestimation of reported practices. Second, the study relied on a convenience sample recruited online, which may introduce selection bias and limit the representativeness of the findings. Third, the relatively small sample size (n = 77) and the short data collection period may further limit generalizability. Fourth, the questionnaire was not formally validated, which may affect the reliability of the measurements. Finally, the cross-sectional design precludes any assessment of causal relationships between knowledge, training, and practices.

## Conclusions

This study highlights a gap between knowledge and clinical practice regarding neonatal transport among healthcare providers in the Oriental region of Morocco. Although participants demonstrated high levels of knowledge on key aspects of neonatal transport, the implementation of recommended practices, particularly in terms of stabilization, monitoring, and thermal protection, remains inconsistent. The low rate of formal training and the limited availability of adequately equipped transport systems may contribute to these findings. These results underscore the need for structured training programs, improved organization of neonatal transport services, and better access to appropriate equipment to enhance the quality and safety of neonatal transport. Such efforts may contribute to reducing preventable neonatal morbidity and mortality in resource-limited settings.

## Figures and Tables

**Figure 1:**
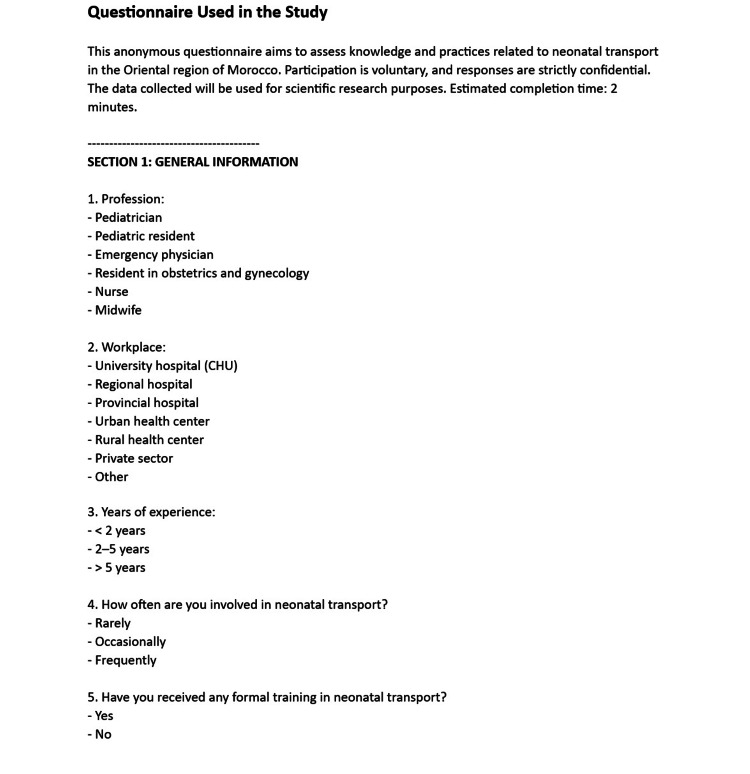
Questionnaire used in the study – Section 1 (General Information)

**Figure 2:**
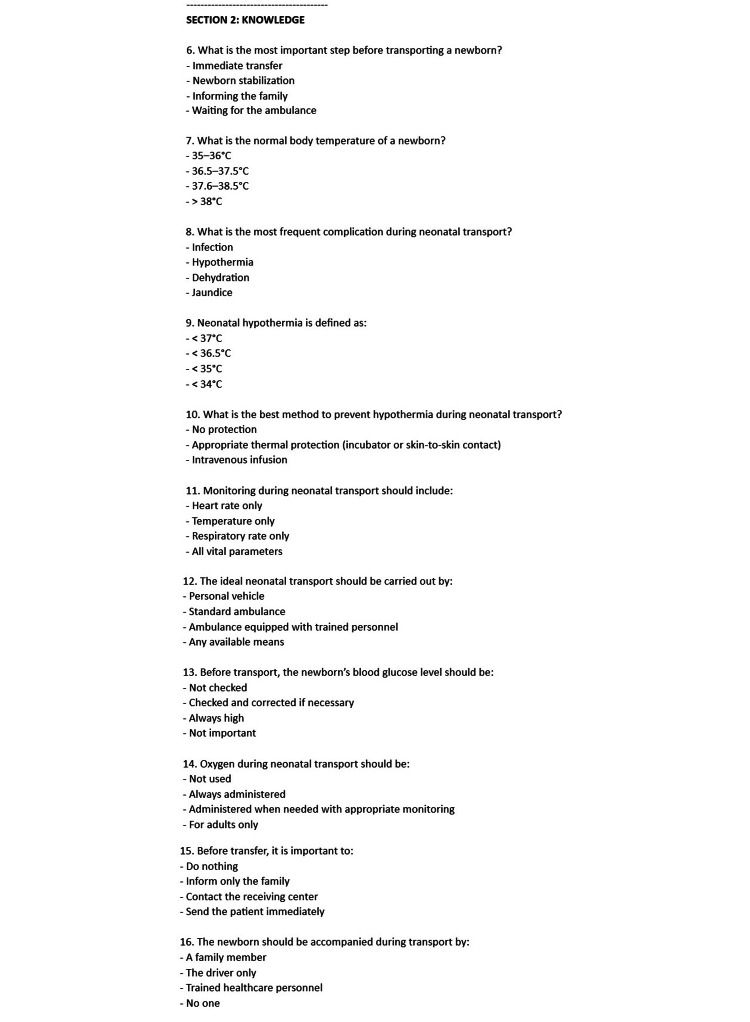
Questionnaire used in the study – Section 2 (Knowledge)

**Figure 3:**
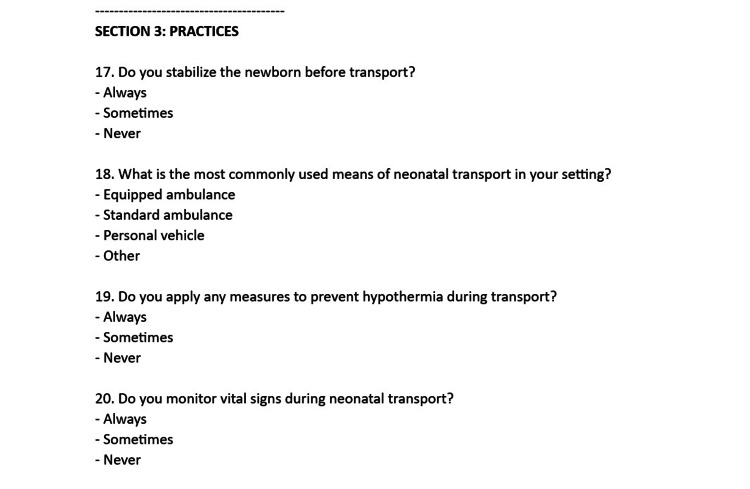
Questionnaire used in the study – Section 3 (Practices)
